# Assessing protein-specific radiation damage in time-resolved X-ray solution-scattering experiments at high-brilliance synchrotrons using fast detector readout

**DOI:** 10.1107/S2059798326005164

**Published:** 2026-06-12

**Authors:** Fatemeh Sabzian-Molaei, Tomás S. Plivelic, Magnus Andersson

**Affiliations:** a Department of Chemistry Umeå University Umeå Sweden; b MAX IV Laborator Lund University Lund Sweden; Institut de Biologie Structurale, France

**Keywords:** time-resolved X-ray solution scattering, small-angle X-ray scattering, radiation damage, adenylate kinase, dose control

## Abstract

Time-resolved X-ray solution scattering (TR-XSS) enables tracking of protein structural dynamics but can suffer from radiation damage when performed with continuous X-ray beam exposure at multipurpose small-angle X-ray scattering (SAXS) beamlines. Here, we systematically assess how protein concentration, exposure time, X-ray dose and beam focusing influence the stability of adenylate kinase (AdK) during TR-XSS at CoSAXS at the MAX IV Laboratory. Static SAXS measurements from 2.5 to 21 mg ml^−1^ showed only minor interparticle effects, establishing suitable concentrations for TR-XSS. Under detector-focused conditions, AdK irradiated at room temperature remained stable up to 3.13 kGy, with consistent absolute and difference scattering profiles over 50 ms. In contrast, focusing the beam directly on the sample increased the absorbed dose to 21.3 kGy and produced clear signatures of radiation damage, which included low-*q* intensity loss and increasing deviation from the low-dose condition. These results identify practical system-specific dose limits and highlight beam focusing as a key determinant of sample integrity. The workflow presented here provides general guidelines for minimizing radiation artifacts in TR-XSS on multipurpose SAXS beamlines.

## Abstract 2

Adenylate kinase was used to quantify how experimental parameters influence radiation damage during time-resolved X-ray solution scattering (TR-XSS) at a multipurpose SAXS beamline. The study provides general guidelines for minimizing radiation artifacts in TR-XSS.

## 1. Introduction

**Figure 1:**
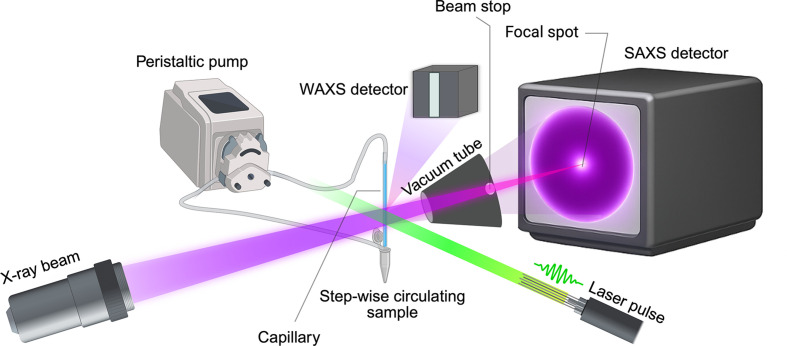
Time-resolved X-ray solution-scattering (TR-XSS) experimental setup with a continuous X-ray beam and rapid-readout SAXS and WAXS detectors. The protein sample was contained in a 0.3 mm capillary and replenished between each 50 ms measurement repeat using a peristaltic pump. A nanosecond UV laser provided photoactivation of caged ATP and a vacuum tube minimized air scattering.

Mapping time-dependent protein structural dynamics is key to understanding functional mechanisms, such as enzyme catalysis, allosteric regulation and membrane transport. These dynamic events frequently involve transient ‘high-energy’ intermediate states that can be difficult to trap and characterize with, for example, X-ray crystallography or cryo-electron microscopy (cryo-EM) techniques. This has created a growing need for methods that can probe protein dynamics at high temporal resolution. Time-resolved X-ray solution scattering (TR-XSS) has emerged as a powerful tool to meet this demand (Sabzian-Molaei *et al.*, 2026 ▸; Pounot *et al.*, 2023 ▸; Cammarata *et al.*, 2008 ▸; Orädd & Andersson, 2021 ▸). In TR-XSS experiments, laser activation can be used to trigger protein dynamics if the protein is sensitive to light, either inherently (Andersson *et al.*, 2009 ▸; Malmerberg *et al.*, 2011 ▸; Berntsson *et al.*, 2017 ▸, 2019 ▸; Takala *et al.*, 2014 ▸; Björling *et al.*, 2016 ▸; Cho *et al.*, 2016 ▸; Malmerberg *et al.*, 2015 ▸; Heyes *et al.*, 2019 ▸; Lee *et al.*, 2022 ▸; Henry *et al.*, 2020 ▸) or by modification, for example by laser-induced release of carbon monoxide bound to hemoglobin or myoglobin (Cammarata *et al.*, 2008 ▸; Kim *et al.*, 2012 ▸, 2020 ▸; Levantino *et al.*, 2012 ▸; Choi *et al.*, 2022 ▸). For most protein targets, such direct activation by light is not possible and instead requires indirect triggering by caged compounds (Ravishankar *et al.*, 2020 ▸; Orädd *et al.*, 2021 ▸; Prabudiansyah *et al.*, 2024 ▸; Magkakis *et al.*, 2024 ▸, 2025 ▸; Josts *et al.*, 2018 ▸), rapid mixing approaches (De Bei *et al.*, 2025 ▸; Tidow & Josts, 2022 ▸; Josts *et al.*, 2020 ▸) or by introducing temperature jumps (Rimmerman *et al.*, 2017 ▸; Thompson *et al.*, 2019 ▸; Chan *et al.*, 2023 ▸; Bennett *et al.*, 2024 ▸; Hsu *et al.*, 2021 ▸). Synchrotron X-rays can then be used to capture protein conformational transitions on the microsecond-to-millisecond timescale (Levantino *et al.*, 2015 ▸; Orädd *et al.*, 2021 ▸). At dedicated time-resolved synchrotron beamlines, mechanical chopper devices are used to select short X-ray pulses, which reduces the risk of radiation damage. TR-XSS characterization of protein dynamics can also be performed with a continuous X-ray beam using detector-readout frequency to obtain temporal resolution (Fig. 1 ▸; Magkakis *et al.*, 2024 ▸; Westenhoff *et al.*, 2010 ▸), which enables the use of, for example, high-brilliance X-rays within an evacuated environment to reduce air scattering and improve the signal-to-noise ratio. However, radiation damage becomes a greater concern in such experimental designs and should be carefully assessed. In TR-XSS experiments, radiation damage is defined as alterations in the difference scattering profiles that are not associated with protein conformational changes, including aggregation and fouling of the sample cell. To distinguish these effects from true protein dynamics, data collections should be compared under identical conditions, for example at the same protein concentration and buffer composition, while varying parameters such as the absorbed dose. In practice, however, limited beamtime allocations often prevent the systematic assessment of radiation damage.

Radiation damage remains a key limitation across all X-ray-based methods, with numerous investigations emphasizing the need to control the absorbed dose and mitigate effects on protein structural integrity (Garman & Weik, 2021 ▸). For example, continuous flow (Fischetti *et al.*, 2003 ▸) or radical scavengers (Castellví *et al.*, 2020 ▸) have been shown to decrease radiation damage in static small-angle X-ray scattering (SAXS) measurements. In the present study, we assess radiation damage in TR-XSS experiments with a continuous X-ray beam by tracking the dynamics of the adenylate kinase (AdK) enzymatic reaction following laser-induced release of ATP at the multipurpose CoSAXS beamline at the MAX IV Laboratory, which hosts versatile capabilities for time-resolved studies (Herranz-Trillo *et al.*, 2024 ▸). AdK is a well characterized phosphotransferase that interconverts adenine nucleotides ATP + AMP ⇌ 2ADP to regulate energy levels in cells (Ionescu, 2019 ▸). The enzymatic reaction involves relocation of the ATP-binding domain (LIDbd) and AMP-binding domain (NMPbd) from an open to a closed conformation, with the two flexible domains attached to the immobile CORE domain to form the catalytic site. The large-scale domain movements and relative ease of protein production make AdK an ideal target for benchmarking and assessment of radiation damage in TR-XSS experiments.

In a previous study, Magkakis *et al.* (2024 ▸) investigated radiation damage during TR-XSS experiments on the AdK enzymatic reaction using a 500 ms continuous X-ray beam with 2 ms detector-readout frequency across 2000 repeats at CoSAXS at the MAX IV Laboratory. This work demonstrated that while single 500 ms exposures did not visibly damage the sample, cumulative damage became apparent in the low-*q* region after multiple exposures. Importantly, it was shown that difference profiles constructed from laser-on minus laser-off scattering profiles effectively canceled out radiation artifacts. These findings provide a foundation for our current study, in which we systematically examine how protein concentration, exposure time and beam-focusing conditions influence radiation effects in TR-XSS experiments.

Elevated protein concentrations can introduce sample artifacts, including aggregation, particle interference and crowding-related conformational biases, which risk obscuring the biological signal of interest. For example, SAXS measurements at increasing protein concentrations were shown to improve scattering intensity but also introduced particle interference that altered measurements of the radius of gyration (*R*_g_), maximum particle dimension (*D*_max_) and forward scattering intensity (*I*_0_) (Jeffries *et al.*, 2016 ▸). In addition, increasing the protein concentration from 8 to 24 mg ml^−1^ in SAXS measurements of cytochrome *c* linearly reduced the relative error across the measured *q* values, although sample aggregation also increased (Sedlak *et al.*, 2017 ▸). Because the effects of protein concentration mainly impact low-*q* regions (Putnam *et al.*, 2007 ▸), SAXS experiments are typically performed below 10 mg ml^−1^ to minimize such distortions. Therefore, characterization of radiation damage at different protein concentrations is particularly important in TR-XSS experiments since short X-ray exposures require high protein concentrations (>10 mg ml^−1^) to obtain sufficient signal-to-noise ratios. In addition, radiation damage may also arise from suboptimal beamline configurations, such as improper beam focusing or excessive beam intensity, which can increase the photon flux density and thereby the absorbed dose, accelerating damage processes, such as protein fragmentation, aggregation or conformational alterations, that might obscure biologically relevant structural changes (Kuwamoto *et al.*, 2004 ▸).

Together, these considerations highlight the need for an experimental framework that directly evaluates how X-ray dose, beam geometry and protein concentration influence sample integrity under TR-XSS conditions. Here, we systematically examine radiation effects on adenylate kinase (AdK) using a combination of static SAXS benchmarking, dose-controlled TR-XSS measurements and comparisons of beam-focusing configurations. By quantifying structural stability across a range of concentrations, exposure times and absorbed dose conditions, we establish practical dose limits and identify beamline settings that minimize radiation artifacts while preserving high-quality time-resolved scattering signals. The resulting procedure provides an operational guideline for assessing radiation damage in TR-XSS measurements on multipurpose SAXS beamlines such as CoSAXS.

## 2. Methods

### 2.1. Small-angle X-ray scattering (SAXS)

Small-angle X-ray scattering (SAXS) experiments were conducted at CoSAXS at the MAX IV Laboratory, Lund, Sweden to probe radiation-damage effects on adenylate kinase (AdK) in solution at varying protein concentrations and X-ray exposure times. Protein samples were prepared at concentrations of 2.5, 5, 10 and 21 mg ml^−1^ as described previously (Orädd *et al.*, 2021 ▸) in a buffer consisting of 30 m*M* MOPS pH 7.0, 50 m*M* NaCl, 2 m*M* MgCl_2_, 2 m*M* AMP. The X-ray beam had a horizontal full width at half maximum (FWHM) of 81.4 µm and an approximately Gaussian intensity profile, with a vertical FWHM of 106.4 µm. Scattering data were collected at two exposure times, 10 and 200 ms, and for each sample 42 frames were recorded, with 0.5 mm spacing between exposures, in a 1.5 mm outer diameter quartz capillary. All measurements were conducted at 21°C. Data processing, including background subtraction, frame averaging and radiation-damage analysis, was carried out using Python tools along with standard SAXS analysis software, including *PRIMUS* (from the *ATSAS* package; Konarev *et al.*, 2003 ▸). Each point in the scattering curves in Fig. 3 corresponds to an average of 42 measurements collected across the capillary. Additional uncertainties arise from the intensity error per detector pixel, averaging of 1000 detector pixels per *q* point and propagation of errors in generating the corrected scattering curve by buffer subtraction. In addition, the magnitude of the error bars varies depending on the beam localization on the four detector panels.

Azimuthal integration was performed using the Python-based azint library at the CoSAXS beamline (available at https://github.com/maxiv-science/azint; Jensen *et al.*, 2022 ▸). Each *q* point represents a weighted average intensity over all contributing pixels within a given *q* bin, calculated as
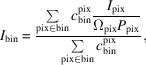
where *I*_pix_ is the raw photon count for the pixel, Ω_pix_ and *P*_pix_ are corrections for solid angle and polarization, respectively, and 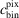 accounts for pixel splitting when pixels partially contribute to a bin.

The associated uncertainty (standard error of the mean) is propagated from the pixel-level Poisson counting statistics using
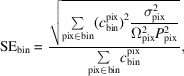
with 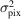 = *I*_pix_ for Poisson statistics. This ensures that consistent account is taken of both geometric corrections and partial pixel contributions in the propagated uncertainty. Consequently, at low sample concentration and short exposure times, lower photon counts per pixel lead to increased Poisson noise and larger propagated uncertainties. Conversely, higher concentrations and longer exposure times result in increased photon counts, reduced statistical noise and smaller standard errors.

### 2.2. Time-resolved X-ray solution scattering (TR-XSS)

Time-resolved X-ray solution-scattering (TR-XSS) experiments were conducted at CoSAXS at the MAX IV Laboratory. A 0.3 mm quartz capillary contained the AdK sample at a concentration of 21 mg ml^−1^ in the same buffer as in the SAXS measurements and including 10 m*M* NPE-caged ATP. The AdK reaction was triggered by a 355 nm nanosecond laser activation of the caged ATP, which in this setup was synchronized with the 4 ms opening of the X-ray shutter. Therefore, at time zero (*t* = 0 s) the reaction had already occurred for 4 ms, after which we tracked the AdK reaction in the series 0–10, 10–20, 20–30, 30–40 and 40–50 ms with a 2 ms frame readout. We repeated the measurements ∼500 times and for each repeat we measured datasets with laser (light) and without laser (dark). For each repeat, the sample was stationary in the capillary. To replenish the sample between measurements, a peristaltic pump was used at a flow rate of 120 µl min^−1^. This corresponds to a linear velocity of 28 mm s^−1^, which during the 1 s pump activity corresponds to a sample displacement of 28 mm, which was substantially larger than the beam dimension (described below), ensuring complete replacement of the previously irradiated volume. Consequently, dose accumulation occurs within individual repeats but not across successive repeats.

#### 2.2.1. Laser excitation and dose control

Activation was triggered using a pulsed nanosecond UV laser focused to a roughly circular spot with an FWHM diameter of approximately 800 µm (∼0.5 mm^2^ area), which was larger than the X-ray dimensions. This design ensured homogeneous photoactivation within the X-ray-probed volume. Furthermore, considering the diffusion constant of free ATP in aqueous solution (∼7.1–7.2 × 10^−6^ cm^2^ s^−1^; Bowen & Martin, 1964 ▸), the released ATP from photolysis remained within the X-ray window for the duration of the longest time point recorded (50 ms), minimizing spatial dilution effects. The laser energy was controlled via the applied voltage and measurements were performed at 1.06 kV, corresponding to an energy density of 5.2 mJ mm^−2^. Data were collected in alternating laser-off/on states ([0, 1]) with delay (4 ms) between activation and measurement, repeated 500 times.

#### 2.2.2. X-ray beam size, focus and dose calculations

Monochromatic X-rays at 12.4 keV (λ = 1 Å) with an energy bandwidth of Δ*E*/*E* = 2 × 10^−4^ were used, delivering a photon flux of approximately 10^1^^2^ photons s^−1^ at the sample position as measured by an ion chamber. The beam was focused at the sample or onto the detector at three different intensities to assess radiation damage. When focused at the detector, the X-ray beam dimensions at the sample were 82.1 × 96.5 µm (∼7923 µm^2^), while focusing at the sample yielded a smaller beam of 39.9 × 29.2 µm (∼1165 µm^2^).

The X-ray dose was calculated using the following equation (Jain *et al.*, 2013 ▸),
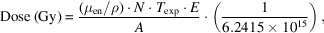
where μ_en_/ρ = 2.5 × 10^8^ µm^2^ g^−1^ is the mass energy-absorption coefficient at 12.4 keV obtained from the NIST database (Seltzer, 1993 ▸) by interpolation between tabulated values, *N* = 10^12^ photons s^−1^ is the photon flux, *T*_exp_ = 0.002 s is the exposure time per frame, *E* = 12.4 keV is the photon energy, *A* is the beam cross-sectional area (in µm^2^; although the X-ray beam has a Gaussian intensity profile, it was approximated as a uniform beam for dose calculations, using the FWHM values to define the effective beam area) and 6.2415 × 10^15^ keV kg^−1^ is a conversion factor to Gy.

Using this approach, the X-ray beam intensities were estimated to be 125.4 Gy (full beam), 62.7 Gy (half beam) and 12.5 Gy (one-tenth beam) for a single 2 ms exposure. In addition, the absorbed dose was calculated using *RADDOSE*-3*D* (Zeldin *et al.*, 2013 ▸; Brooks-Bartlett *et al.*, 2017 ▸) for comparison; the results under different beam conditions are summarized in Supplementary Table S2. The results obtained from the dose equation were in good agreement with those obtained using* RADDOSE*-3*D*. X-ray beam intensity was controlled using aluminium filters installed in the beamline:

(i) *I*_0_ (monitor beam intensity): *I*_0_ = 0.925 ± 0.002.
(ii) *I*_0_-AL180 (intensity after 180 µm aluminium filter): *I*_0_-Al180 = 0.463 ± 0.002 (transmission ≃ 50%).
(iii) *I*_0_-AL540 (intensity after 540 µm aluminium filter): *I*_0_-Al540 = 0.1145 ± 0.0008 (transmission ≃ 12.4%).

#### 2.2.3. Detector configuration and scattering ranges

Scattered X-rays were recorded simultaneously in the SAXS and WAXS regimes. SAXS data were collected using an EIGER2 4M (Dectris) detector placed 2.08 m from the sample in vacuum, covering a *q*-range of 0.01–0.6 Å^−1^. WAXS data were collected using a MYTHEN2 1K (Dectris) detector positioned 0.253 m from the sample in air, spanning *q* = 0.9–2.5 Å^−1^. The solvent heating response was measured in the WAXS *q*-range 1.3–2.3 Å^−1^, with laser-off/laser-on difference profiles obtained by subtracting the average scattering signal over 0–50 ms, calculated from 500 repeats (Supplementary Fig. S4). The frame rate of 500 Hz on the EIGER2 detector set the effective temporal resolution to 2 ms per frame. Following background subtraction, laser-on and laser-off profiles were averaged separately and the difference signal Δ*S*(*q*) was computed for each delay condition.

#### 2.2.4. Determination of radius of gyration (*R*_g,app_) and maximum particle dimension (*D*_max_)

To characterize the protein structure and assess the feasibility of time-resolved experiments, SAXS data were first analyzed using Guinier plots to examine concentration-dependent effects. Only the linear regions of the Guinier plots were considered in order to minimize contributions from interparticle interactions, and the corresponding *q*-ranges were used for subsequent analyses. From these *q*-ranges, pair-distance distribution functions, *P*(*r*), were calculated using *GNOM* (v.5.0, r14288) from the *ATSAS* package v.3.1.0 (Manalastas-Cantos *et al.*, 2021 ▸). The function *P*(*r*) represents the probability distribution of distances between scattering points within the particle and provides information about its overall size and shape. The chosen *q*-ranges resulted in acceptable solutions according to the *GNOM* quality metric, with total estimate values ≥ 0.7 for all datasets.

The apparent radii of gyration (*R*_g,app_) were then obtained from *P*(*r*) as the root-mean-square distance of scattering points from the particle’s center of mass,
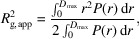
where the maximum particle dimension (*D*_max_) was defined as the distance at which *P*(*r*) approaches zero, representing the largest intraparticle separation. The *D*_max_ value was estimated by extrapolation in the Guinier plot and optimized to produce acceptable fits to the experimental scattering data. The uncertainties in *R*_g,app_ were estimated from the *GNOM* calculations and reflect the sensitivity of the calculated mean-square distance to small variations in *P*(*r*) within the fitting uncertainty.

#### 2.2.5. Singular value decomposition and extraction of basis spectra

The time-resolved difference scattering curves, Δ*S*(*q*, *t*), were arranged into a matrix with dimensions corresponding to the number of *q* values and time delays. Singular value decomposition (SVD) was first applied to evaluate the number of significant components contributing to the dataset. Inspection of the singular values and the autocorrelation of the singular vectors indicated that two dominant components describe the TR-XSS signal (Orädd *et al.*, 2021 ▸). To extract kinetic information from the SVD basis spectra, the data were then fitted using a sequential two-state model describing the formation of a transient state upon laser excitation. The model assumes a single transition from an early state to a late state following first-order kinetics:
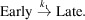
Global fitting was employed to extract the rate constant and the time-dependent basis spectra, based on
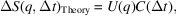
where *U*(*q*) is a matrix containing the static basis spectra extracted from SVD and *C*(Δ*t*) represents the time-dependent concentration of the spectral components were obtained by integrating the corresponding rate equations:
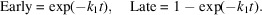


#### 2.2.6. *R*-factor calculation

To quantify the divergence between the high-dose and low-dose datasets, we calculated *R* factors between the corresponding difference scattering profiles. For each 10 ms time interval, the *R* factor was computed according to
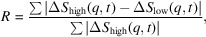
where Δ*S*_high_(*q*, *t*) and Δ*S*_low_(*q*, *t*) represent the difference scattering intensities obtained under high-dose and low-dose beam conditions, respectively.

## 3. Results

### 3.1. Effects of protein concentration and exposure time on SAXS profiles

**Figure 2:**
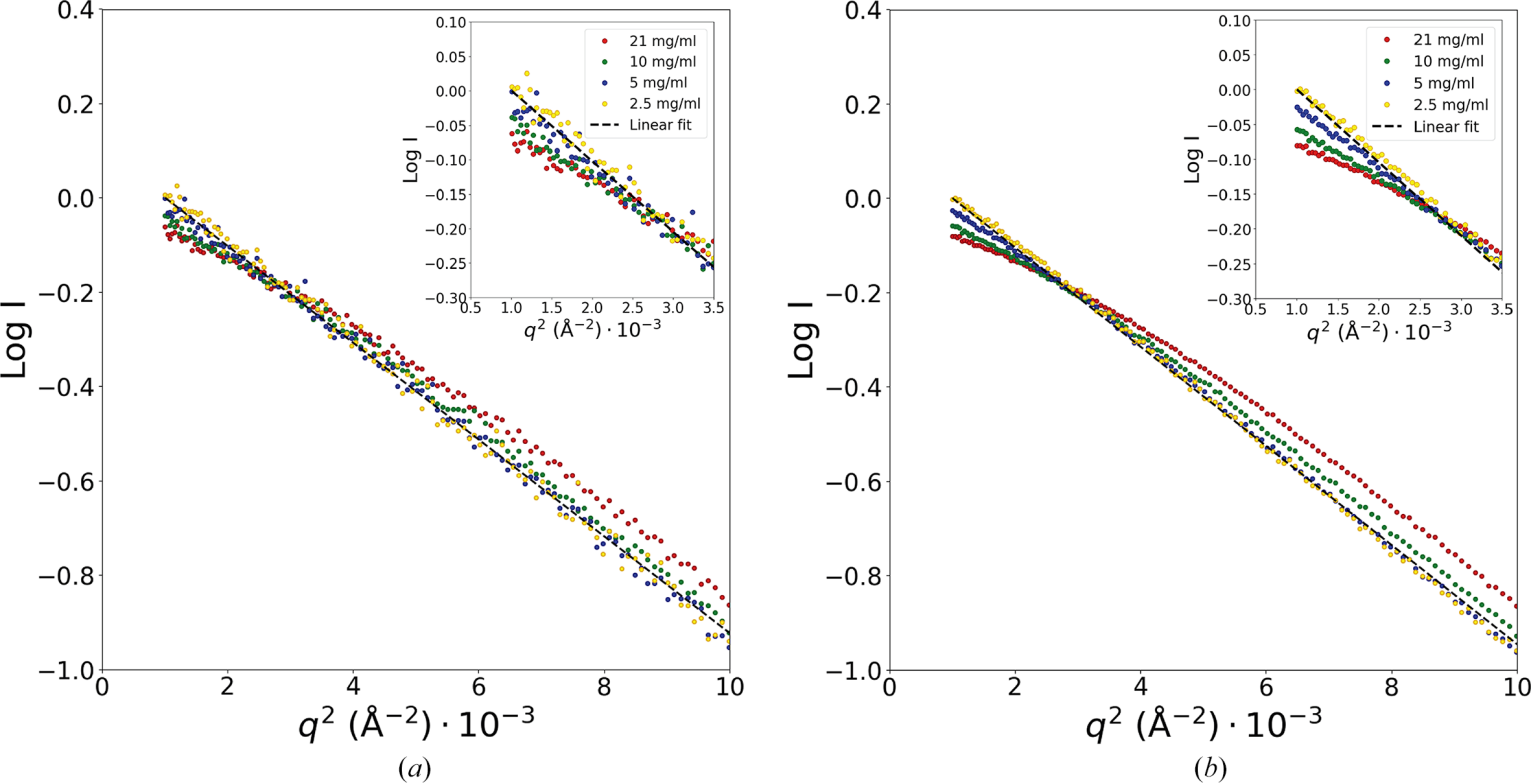
Assessing particle interactions in AdK samples at different protein concentrations and exposure times. Guinier plots at protein concentrations of 2.5 mg ml^−1^ (yellow), 5 mg ml^−1^ (blue), 10 mg ml^−1^ (green) and 21 mg ml^−1^ (red) were collected at (*a*) 10 ms and (*b*) 200 ms exposure times. The dashed line represents a linear fit to the 2.5 mg ml^−1^ data.

To assess the effects of X-ray radiation at different protein concentrations, SAXS data were collected at AdK protein concentrations of 2.5, 5, 10 and 21 mg ml^−1^ during 10 and 200 ms X-ray exposures. Absolute scattering curves *I*(*q*) were averaged from 42 frames recorded at 0.5 mm intervals along a quartz capillary with 1.5 mm outer diameter. To evaluate concentration-dependent effects, Guinier plots were generated (Fig. 2 ▸). At 2.5 mg ml^−1^ protein concentration, a linear plot was obtained which corresponds to dilute conditions without significant particle interference. With increasing protein concentration, a progressively stronger downward curvature was observed at low *q*, indicating small but detectable repulsive particle interactions.

**Figure 3:**
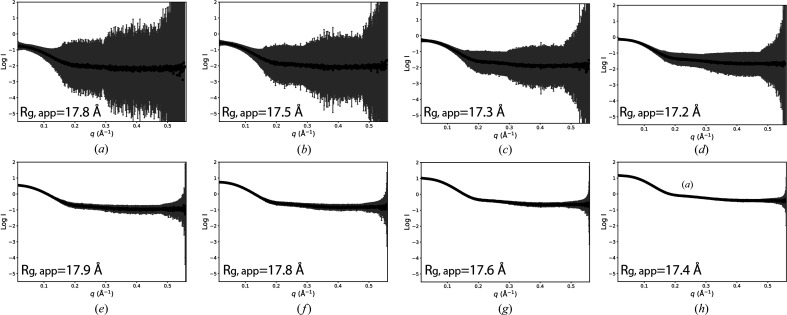
Effects of AdK protein concentration and X-ray exposure on absolute scattering intensity. Intensity [*I*(*q*)] as a function of scattering vector (*q*) at 10 ms exposure for concentrations of (*a*) 2.5 mg ml^−1^, (*b*) 5 mg ml^−1^, (*c*) 10 mg ml^−1^ and (*d*) 21 mg ml^−1^, and similarly at 200 ms exposure for (*e*) 2.5 mg ml^−1^, (*f*) 5 mg ml^−1^, (*g*) 10 mg ml^−1^ and (*h*) 21 mg ml^−1^. Gray-shaded regions represent standard error bars, with their origins explained in Section 2.

We then calculated pair-distance distribution functions, *P*(*r*), based on *q*-ranges selected after excluding the low-*q* region (Supplementary Table S1) to minimize particle interaction effects and optimization of the *GNOM* fit quality (Supplementary Fig. S1). Although this approach largely reduced the influence of particle interactions, a minor concentration-dependent trend remained. From these *P*(*r*) functions, we obtained an apparent radius of gyration (*R*_g,app_) that showed minor variations across the concentration range (Supplementary Table S1). The slight decreases in *R*_g,app_ and *D*_max_ with increasing protein concentration are consistent with weak repulsive interactions in solution. Furthermore, extending the exposure time from 10 to 200 ms resulted in a similarly small increase in *R*_g,app_ (<0.5 Å; Fig. 3 ▸ and Supplementary Table S1). Therefore, although a 2.5 mg ml^−1^ protein concentration would be optimal to minimize interparticle interactions, the relatively small changes observed in *P*(*r*) and *R*_g,app_ suggest that higher concentrations and exposure times are still feasible for time-resolved experiments. For TR-XSS experiments, such information is important since high protein concentrations and numerous repetitive short exposure times are typically required to obtain sufficient signal-to-noise ratios.

### 3.2. Effect of beam focusing on radiation damage

**Figure 4:**
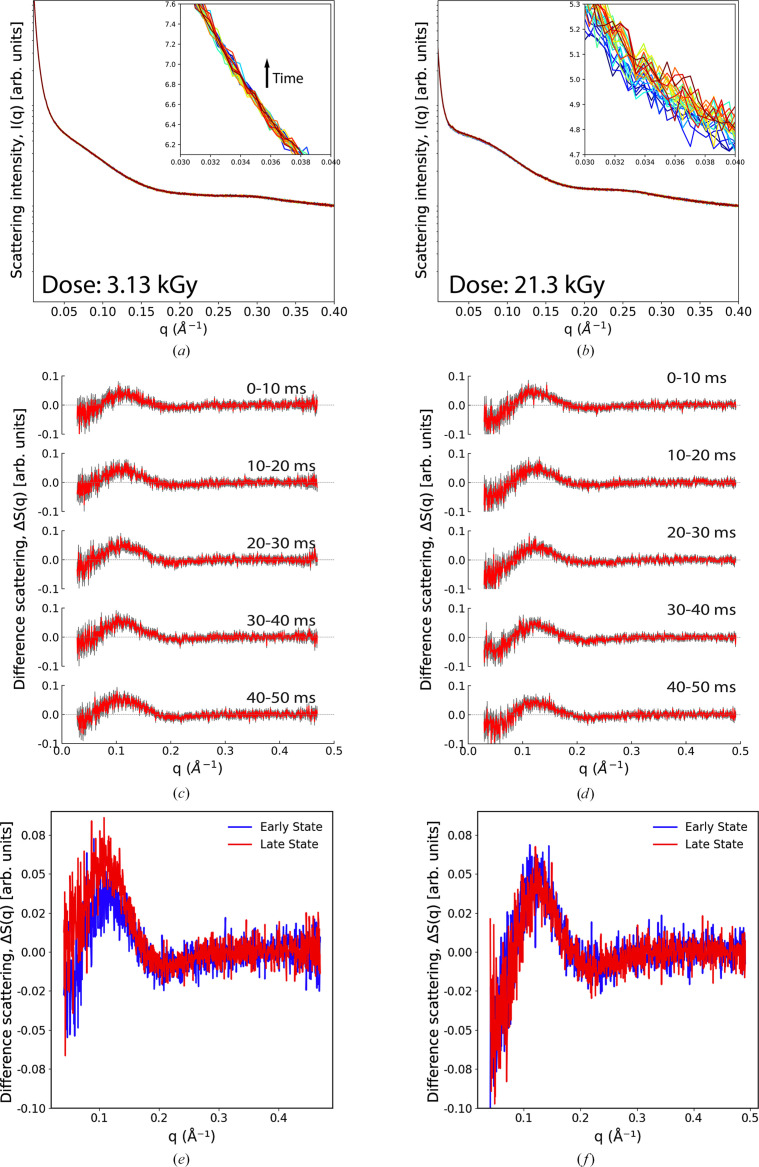
Adenylate kinase difference and absolute scattering profiles. Averaged absolute scattering intensity curves as a function of the scattering vector *q* for (*a*) 3.13 kGy (detector-focused beam) and (*b*) 21.3 kGy (sample-focused beam). Difference scattering profiles averaged in 10 ms intervals from 0 to 50 ms at absorbed X-ray doses of (*c*) 3.13 kGy and (*d*) 21.3 kGy. Time-independent basis spectra obtained from TR-XSS kinetic modeling show early (blue) and late (red) states of the AdK reaction over 50 ms with the beam focused on (*e*) the detector and on (*f*) the sample.

In many TR-XSS experimental setups, the X-ray beam is typically focused slightly before or after the sample to maximize the scattering signal. Changing the beam focus alters the beam size at the sample, which in turn changes the flux density, as described in Section 2. Such variations in flux can lead to elevated absorbed doses and potential radiation damage. To evaluate the effect of X-ray beam focusing, we compared two TR-XSS configurations: one where the X-ray beam was focused onto the sample (21.3 kGy) and a modified setup where the X-ray beam was instead focused on the detector (3.13 kGy). Indeed, the absolute scattering profiles from the laser-off (dark) measurements differed substantially between the two conditions. In the low-dose condition with the beam focused on the detector, low-*q* intensity curves remained stable (Fig. 4 ▸*a*), compared with the high-dose condition with the beam focused onto the sample, which showed greater variance over time (Fig. 4 ▸*b*). Thus, there is a clear dose-dependence that can be observed in the scattering profiles when X-ray beam focusing increases the absorbed energy deposition.

The resulting TR-XSS difference scattering curves contained a positive peak slightly above 0.1 Å^−1^ in both dose conditions (Figs. 4 ▸*c* and 4 ▸*d*), which is a typical AdK protein signal observed in previous TR-XSS experiments (Magkakis *et al.*, 2024 ▸, 2025 ▸; Orädd *et al.*, 2021 ▸). The observed difference features did not display similar time-dependent changes in amplitude compared with earlier AdK TR-XSS experiments (Magkakis *et al.*, 2024 ▸, 2025 ▸; Orädd *et al.*, 2021 ▸). One reason is that in the present study we collected ∼75% fewer data since the purpose was to compare conditions to detect radiation damage, rather than extracting high-quality data for downstream structural interpretation. In addition, due to a 4 ms dead time after laser activation (see Section 2), we are probing a temporal regime with much less pronounced differences (see, for example, Orädd *et al.*, 2021 ▸). Therefore, to assess possible variations in the time-resolved spectra building up over time, which is typical for radiation-damage effects, we used a two-state sequential kinetic model to decompose the data. Comparing the resulting time-independent basis spectra collected under the low-dose condition, a significant increase in amplitude could be observed in the late state compared with the early state (Fig. 4 ▸*c*). This amplitude increase of the 0.1 Å^−1^ positive peak is a signature of the AdK enzymatic reaction and has been monitored in previous TR-XSS experiments (Magkakis *et al.*, 2024 ▸, 2025 ▸; Orädd *et al.*, 2021 ▸). However, at the high-dose condition this amplitude increase was absent (Fig. 4 ▸*d*), indicating that radiation damage becomes more pronounced at later time points (Supplementary Fig. S2).

**Figure 5:**
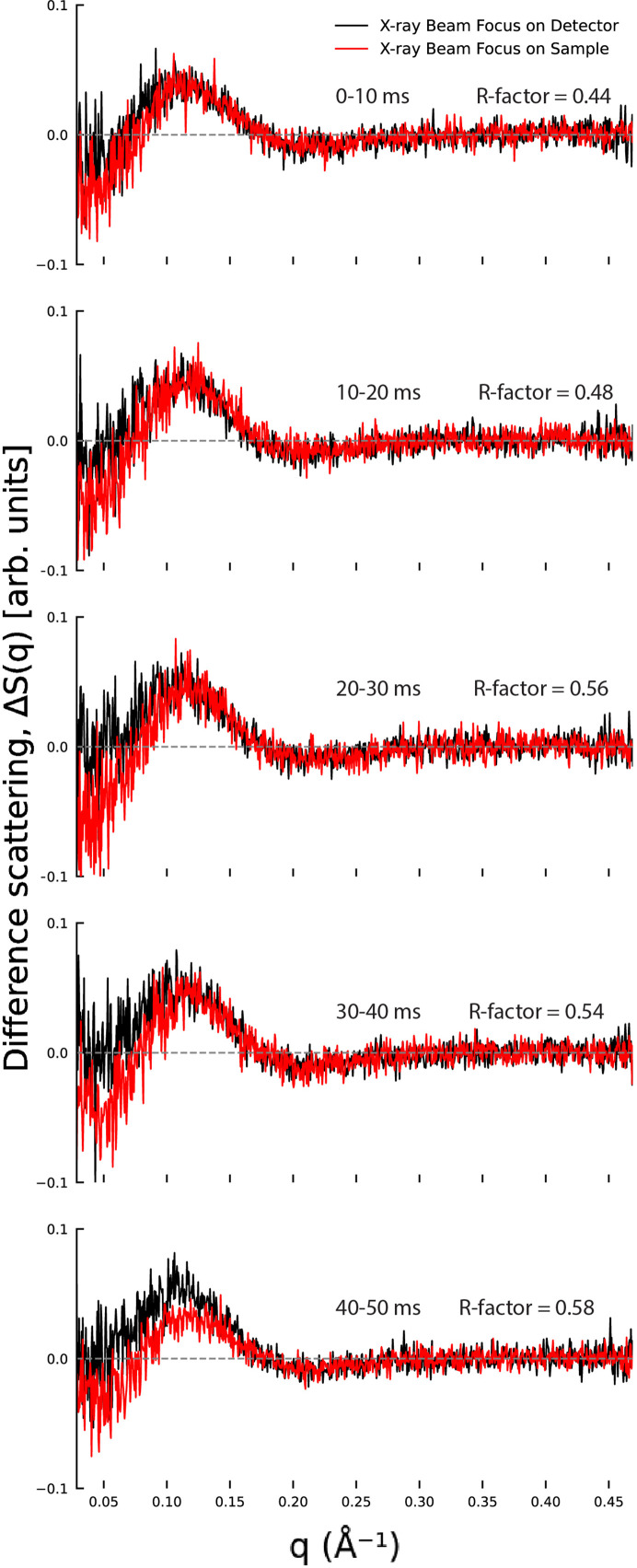
Quantitative comparison of difference scattering profiles at low and high doses. AdK TR-XSS data are shown averaged in 10 ms intervals from 0 to 50 ms at 3.13 kGy dose with the beam focused on the detector (black) and at 21.3 kGy dose with the beam focused on the sample (red). The displayed *R* factors were calculated between the two curves for each time interval.

This trend is further substantiated by tracking the similarity of the difference scattering curves over successive 10 ms intervals (Fig. 5 ▸). An *R*-factor analysis (see Section 2) showed that while the 0–10 ms time intervals were comparable for both conditions, the obtained *R* factors increased steadily over time, reaching maximum values at the final time delay of 40–50 ms. In particular, the high-dose curves showed an increasing negative feature below 0.1 Å^−1^ and also a reduced amplitude in the 0.12 Å^−1^ positive peak. Thus, the *R*-factor metric provides a valuable quantitative assessment of radiation damage when comparing, for example, different experimental designs.

### 3.3. Dose dependence with the beam focused on the detector

**Figure 6:**
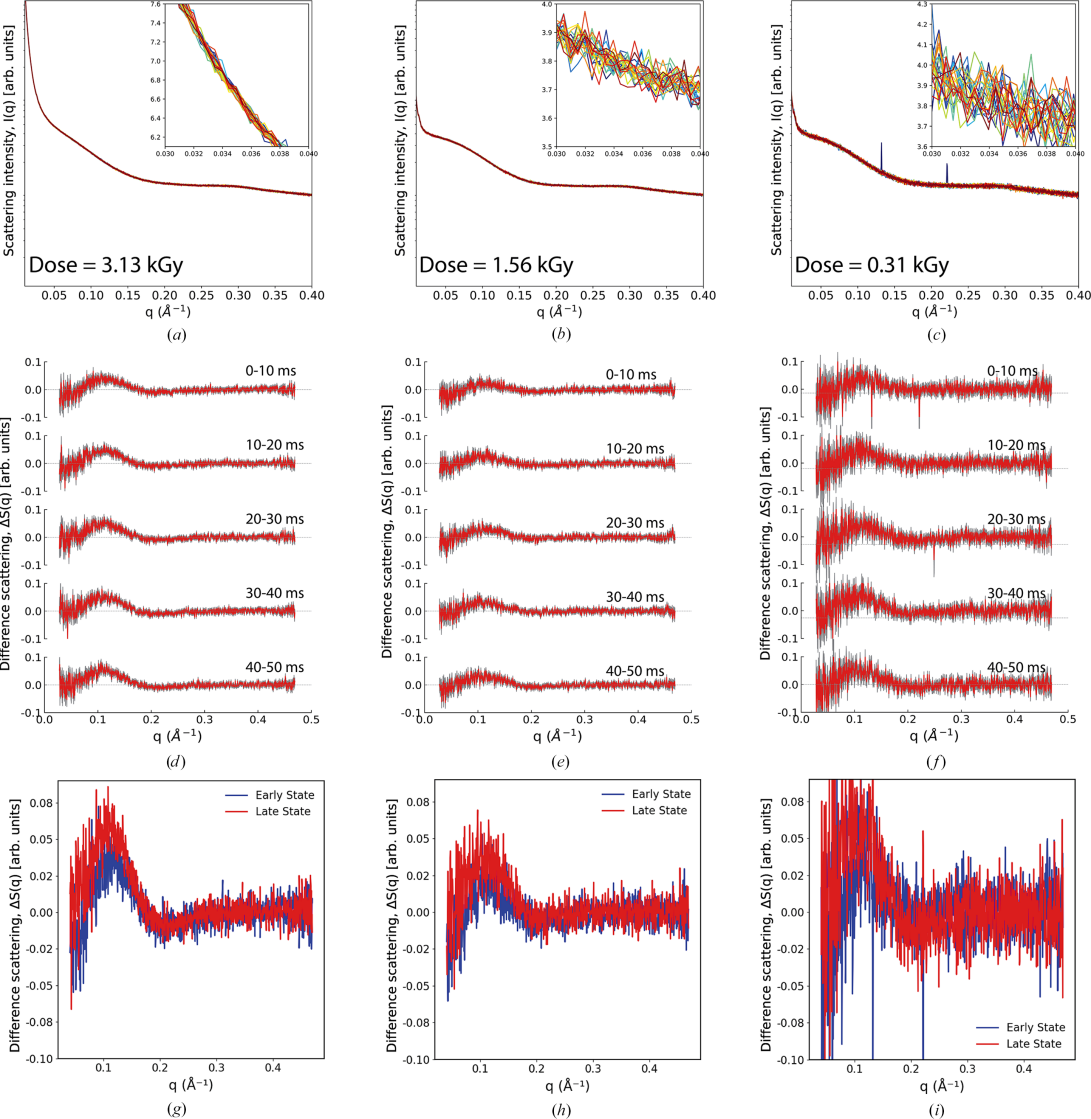
Effects of the absorbed X-ray dose. AdK averaged absolute scattering intensity curves for X-ray beam doses of (*a*) 3.13 kGy, (*b*) 1.56 kGy and (*c*) 0.31 kGy. Difference profiles averaged in 10 ms intervals from 0 to 50 ms at absorbed X-ray doses of (*d*) 3.13 kGy, (*e*) 1.56 kGy and (*f*) 0.31 kGy. The corresponding basis spectra obtained from kinetic modeling are shown at (*g*) 3.13 kGy, (*h*) 1.56 kGy and (*i*) 0.31 kGy.

To further examine radiation-damage effects with X-rays focused on the detector, AdK samples were exposed to accumulated doses of 3.13, 1.56 and 0.31 kGy during a total time of 50 ms. The absolute scattering profiles remained stable over time, with no emerging low-*q* deviations (Figs. 6 ▸*a*–6 ▸*c*). In addition, the corresponding difference scattering profiles, averaged over 10 ms intervals, evolved with a similar pattern across the time series (Figs. 6 ▸*d*–6 ▸*f*), which confirms that under these conditions radiation-induced structural perturbations were negligible. We also calculated time-independent basis spectra and observed that the amplitude of the late-state basis spectra decreased as the beam intensity was reduced from full beam to half beam, accompanied by increased noise, indicating reduced data quality. At one-tenth beam intensity, the spectra suffered from further reduction in the signal to noise, making reliable interpretation difficult (Figs. 6 ▸*g*–6 ▸*i*). These results indicate that using the full beam, with the focus positioned at the detector, provides the optimal balance between minimizing radiation damage and maintaining adequate scattering statistics.

## 4. Discussion

Understanding how X-ray radiation affects protein integrity is essential for reliable TR-XSS measurements, particularly on beamlines that lack X-ray choppers and rely on continuous exposures. Although SAXS dose limits have been estimated previously (Kirby *et al.*, 2016 ▸; Brooks-Bartlett *et al.*, 2017 ▸; Hopkins & Thorne, 2016 ▸), no peer-reviewed study that we are aware of has systematically evaluated radiation damage under TR-XSS conditions involving fast detector readout and laser activation. To address this gap, we examined how protein concentration, exposure time, beam intensity and beam focusing influence the structural stability of AdK during TR-XSS at CoSAXS at the MAX IV Laboratory.

### 4.1. Concentration-dependent static SAXS establishes a baseline for TR-XSS conditions

We first measured static SAXS profiles of AdK at 2.5–21 mg ml^−1^ to evaluate interparticle effects at concentrations relevant for TR-XSS experiments. Increasing concentration produced only modest downward curvature in Guinier plots, consistent with weak repulsive interactions (Sedlak *et al.*, 2017 ▸; Jeffries *et al.*, 2016 ▸). Such effects can be reduced by increasing the ionic strength by, for example, salt addition, changing the buffering system or including stabilizing additives. In this study, however, the buffer (30 m*M* MOPS pH 7.0, 50 m*M* NaCl, 2 m*M* MgCl_2_, 2 m*M* AMP) was chosen to keep adenylate kinase in a ligand-bound, functionally relevant state. Both Mg^2+^ and AMP are important for stabilizing the native conformational ensemble, and changing the salt concentration or buffer composition could influence electrostatic screening, ligand binding or the conformational equilibrium of the protein. We therefore proceeded with the highest concentration as a reasonable compromise between minimizing interparticle effects and achieving sufficient signal to noise. In addition, weak repulsive interactions generally have limited impact on TR-XSS difference curves, where interparticle contributions largely cancel. Nevertheless, extremely high concentrations may restrict protein dynamics, emphasizing the importance of concentration benchmarking prior to TR-XSS. Hence, the concentration series was used as an assessment of sample quality and signal robustness rather than as a quantitative analysis of interparticle interactions.

### 4.2. Operational modes of TR-XSS and implications for radiation exposure

Two experimental strategies are commonly used to achieve time resolution in TR-XSS. Pump-and-probe setups use isolated X-ray pulses synchronized with laser activation (Cammarata *et al.*, 2009 ▸), whereas detector-based approaches exploit rapid detector readout during continuous X-ray exposure (Westenhoff *et al.*, 2010 ▸; Magkakis *et al.*, 2024 ▸). The latter enables TR-XSS on multipurpose SAXS beamlines, such as CoSAXS at the MAX IV Laboratory, but exposes the sample to uninterrupted radiation, which increases the risk of radiation damage. Dedicated time-resolved beamlines mitigate this with X-ray choppers, which are typically not available at multipurpose beamlines. Therefore, assessment strategies such as those presented here are advised in continuous-beam experiments with fast-readout detectors.

### 4.3. Beam focusing strongly determines the absorbed dose and radiation tolerance

Our experiments show that AdK remains structurally stable up to an accumulated dose of 3.13 kGy when the X-ray beam is focused on the detector, and thereby distributing the flux over a larger sample volume. Under these conditions, difference and absolute scattering profiles remained highly consistent over the 50 ms time window. In contrast, focusing the beam directly on the sample increased the absorbed dose to 21.3 kGy and produced clear signatures of radiation damage, which includes a progressive loss of low-*q* intensity and increasing deviation from the corresponding low-dose condition. These results show that beam focusing is important for determining sample stability. This is an especially relevant point for beamlines with adjustable focusing optics, where small positional shifts can substantially alter the absorbed energy deposition.

### 4.4. General implications for TR-XSS experiment design

Although our study focuses on the AdK enzyme, the combined approach of static SAXS benchmarking, dose-controlled TR-XSS and comparison of beam geometries provides a framework for assessing radiation damage in TR-XSS experiments. In solution, radiation damage is dominated by indirect effects from water radiolysis induced by ionizing X-ray radiation rather than direct X-ray interactions with the protein (Houée-Levin & Sicard-Roselli, 2001 ▸). Hence, X-ray absorption by the solvent generates reactive species, such as hydroxyl radicals and solvated electrons, which subsequently modify protein structure and the observed scattering signals. The extent and kinetics of these radical-mediated processes depend strongly on buffer composition, scavenging capacity, protein stability, absorbed dose and the sensitivity of specific conformational states or reaction intermediates. In addition, radiation tolerance varies widely among proteins and depends on molecular size, shape, oligomeric state, hydration shell, intrinsic flexibility, chemical composition and buffer conditions. Therefore, system-specific validation, such as that in the reported study, is required rather than relying on general dose estimates.

### 4.5. Recommendations for TR-XSS experiments on general-purpose SAXS beamlines

Based on our findings, we recommend that TR-XSS experiments (i) benchmark concentration-dependent scattering under static conditions to identify crowding effects and suitable working conditions, (ii) quantify the absorbed dose for the actual beam geometry since beam focusing strongly modulates it, (iii) use detector-focused geometries whenever possible to reduce the absorbed dose within the illuminated sample area without compromising the protein signal and (iv) validate sample stability through time-resolved difference profiles so that radiation effects are not misinterpreted as biologically relevant dynamics. Together, these guidelines define a practical, system-dependent framework for minimizing radiation artifacts and ensuring robust TR-XSS measurements on multipurpose SAXS beamlines.

## Supplementary Material

Supplementary Tables and Figures. DOI: 10.1107/S2059798326005164/xh5064sup1.pdf

## Data Availability

All data and scripts are available upon request.
