# Supplementary material for: Assessing protein-specific radiation damage in time-resolved X-ray solution-scattering experiments at high-brilliance synchrotrons using fast detector readout
Source: Acta Crystallogr D Struct Biol. 2026 Jun 12;82(Pt 7):715–26. doi: 10.1107/S2059798326005164 (PMC13317681; doi:10.1107/S2059798326005164)
Supplement: Supplementary file 1 [file d-82-00715-sup1.pdf]

Acta Cryst D STRUCTURAL BIOLOGY

Volume 82 (2026)

Supporting information for article:

Assessing protein-specific radiation damage in time-resolved X-ray solution-scattering experiments at high-brilliance synchrotrons using fast detector readout

Fatemeh Sabzian-Molaei, Tomás S. Plivelic and Magnus Andersson

Table S1 Radius of gyration (Rg, app), maximum particle dimension (Dmax), and q-range used for P(r) calculation obtained from SAXS data at different protein concentrations and delay times (10 ms and 200 ms).

Concentration (mg/mL)	Time (ms)	Rg, app (Å)	Dmax (Å)	q-range for P(r)
2.5	10	17.8 ± 1.44	52.6 ± 4.28	0.014 - 0.37
5	10	17.5 ± 0 .91	50.6 ± 2.64	0.028 - 0.37
10	10	17.3 ± 0 .72	49.1 ± 2.06	0.050 - 0.37
21	10	17.2 ± 0 .40	48.1 ± 1.10	0.056 - 0.37
2.5	200	17.9 ± 0.15	53.0 ± 0.46	0.014 - 0.37
5	200	17.8 ± 0.01	51.7 ± 0.03	0.028 - 0.370
10	200	17.6 ± 0.01	51.0 ± 0.03	0.050 - 0.370
21	200	17.4 ± 0.04	50.8 ± 0.14	0.056 - 0.370

Table S2 Radiation doses under different beam conditions. Average dose (exposed region) is calculated using the method reported in the manuscript and compared with RADDOSE-3D (absorbed dose).

**Beam conditions**	**Full beam (Sample-focused setup)**	**Full beam (detector-focused setup)**	**Half beam (detector-focused setup)**	**1/10 beam (detector-focused setup)**	**Description**
**Average dose (exposed region), from our calculation**	21.3 kGy	3.13 kGy	1.56 kGy	0.31 kGy	From the equation (reported in the manuscript): $\text{Dose (Gy)} = \frac{(\frac{\mu_{en}}{\rho}) . \text{N} . \text{T}_{exp} . \text{E}}{\text{A}} . (\frac{1}{6.2415 \times 10^{15}})$
**Average dose (exposed region), from RADDOSE-3D**	19.4 kGy	2.88 kGy	1.44 kGy	0.28 kGy	Average dose where the beam hits the sample

The following input parameters were used to run RADDOSE-3D for a full beam (detector-focused setup) with an exposure time of 50 ms.This example can be run directly using the RADDOSE-3D web server interface.

```
##############################################################################
#  Crystal Block ("Crystal" is still used but we are defining a SAXS sample) #
##############################################################################
Crystal
Type Cylinder
Dimensions 100 280             #length × diameter in µm.
PixelsPerMicron 0.5            # The computational resolution
CoefCalc  SAXSseq
SeqFile 4OR0.fasta
ProteinConc 21
ContainerMaterialType elemental
MaterialElements Si 1 O 2
ContainerThickness 10
ContainerDensity 2.2

##############################################################################
#                                  Beam Block                                #
##############################################################################
Beam
Type Gaussian
Flux 1e12
FWHM 82.1 96.5                # µm
Energy 12.4                   # KeV
Collimation Rectangular 82.1 96.5

##############################################################################
#                                  Wedge Block                               #
##############################################################################
Wedge 0 0                     # single orientation
ExposureTime 0.05            #second
# AngularResolution 2
```

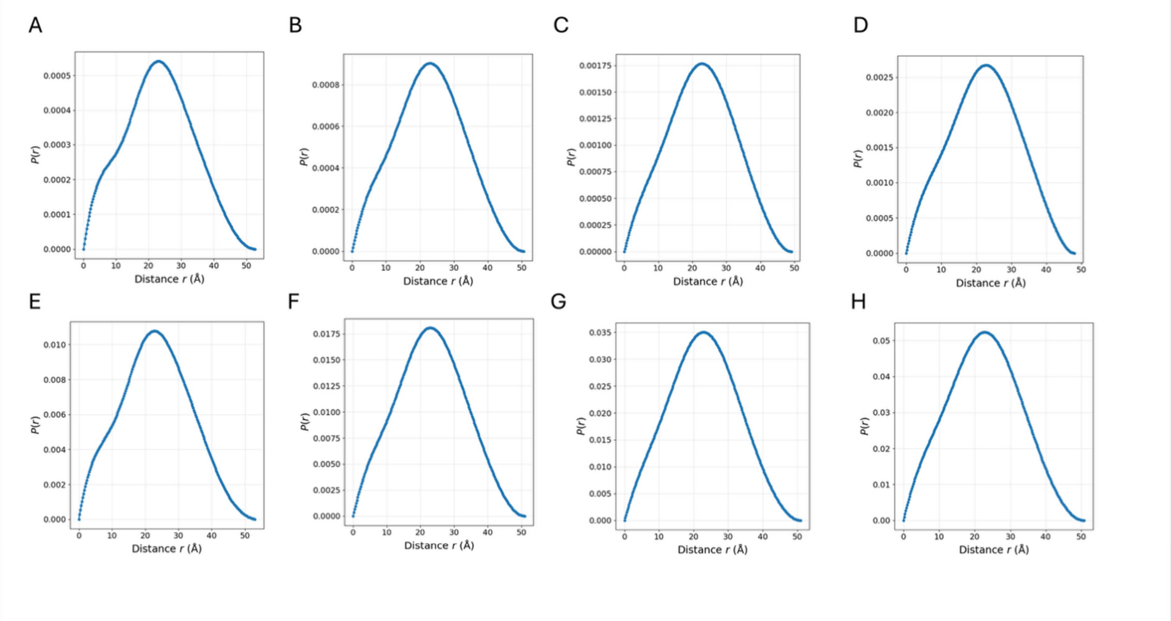

Figure S1 Pair distance distribution functions, P(r), obtained from SAXS for protein concentrations of 2.5, 5, 10, and 21 mg/mL at delay times of (A-D) 10 ms and (A-D) 200 ms . The corresponding P(r) functions were used to determine the radius of gyration (Rg) and maximum particle dimension (Dmax).

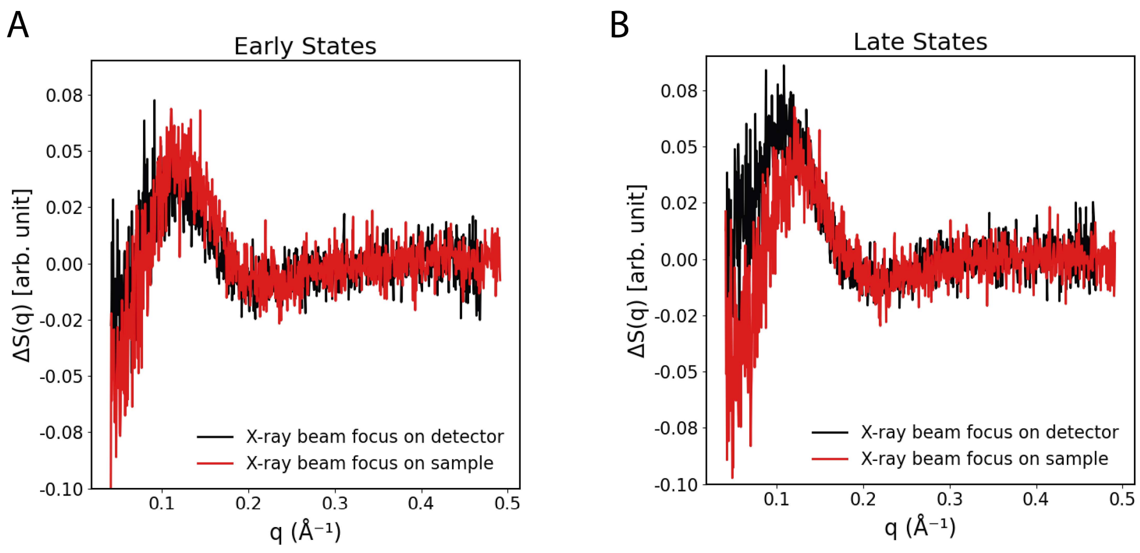

Figure S2 Basis spectra obtained from kinetic modeling **(A)** Early-state for the two experimental configurations: X-ray beam focused on the detector (black) and X-ray beam focused on the sample (red). **(B)** Late-state for the same configurations: detector focus (black) and sample focus (red).

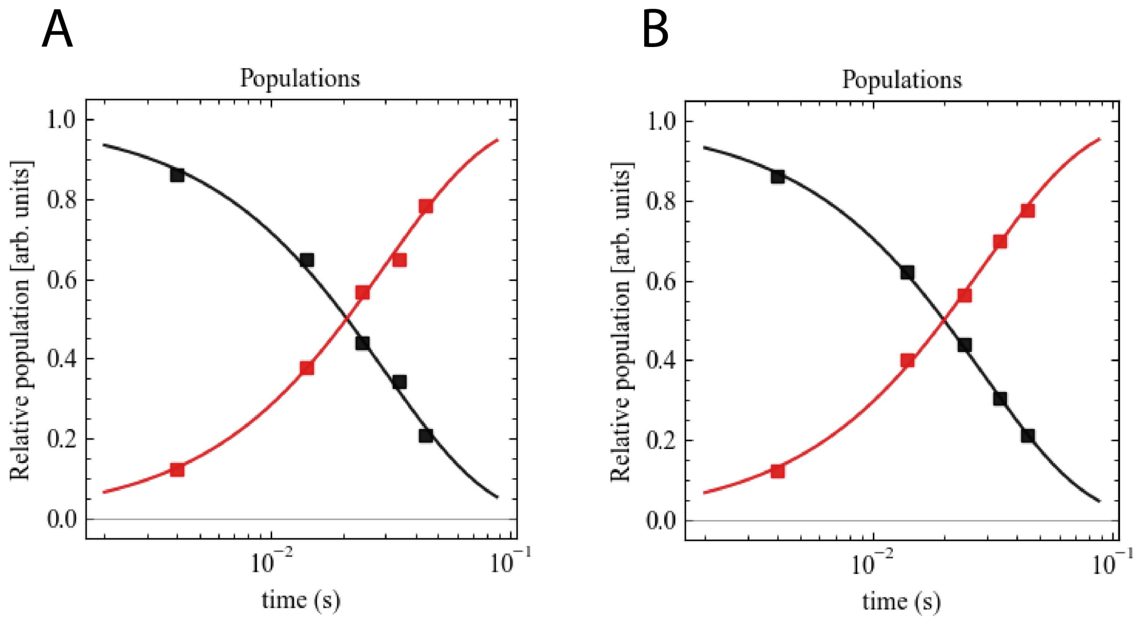

Figure S3 Time-dependent evolution of the relative population of the two kinetic states across experimental time points are shown for **(A)** 22.5 kGy and **(B)** 3.31 kGy.

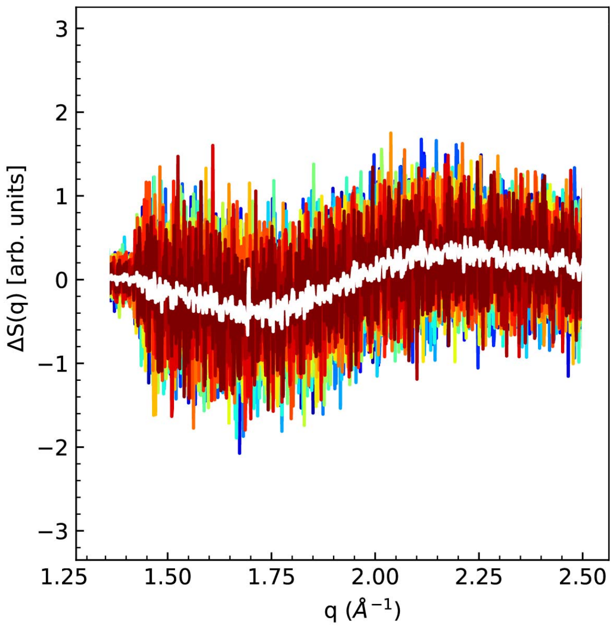

Figure S4 Heating response of the solvent measured by the WAXS detector in the q-range 1.3 $Å^{-1} < q <$ 2.3 $Å^{-1}$. Laser-off/laser-on difference profiles were obtained by subtracting the average scattering signal over 0-50 ms, calculated from 500 repeats.
